# The Relationship Between the NSP and the Individual and Work Organizational Variables: A Cross-Sectional Study

**DOI:** 10.3389/fpubh.2022.726826

**Published:** 2022-03-31

**Authors:** Sue Yuan, Yunxia Li, Lihui Zhang, Honghong Wang

**Affiliations:** ^1^Teaching and Research Section of Clinical Nursing, Xiangya Hospital of Central South University, Changsha, China; ^2^Xiangya Nursing School, Central South University, Changsha, China

**Keywords:** neck-shoulder pain, healthcare workers, occupational health, work-related musculoskeletal disorders, neck disability index

## Abstract

**Objectives:**

To investigate the characteristics of neck–shoulder pain (NSP) and explore the potentional relationship between the NSP and the individual and work organizational variables.

**Methods:**

A cross-sectional study was performed in the tertiary general hospitals in Hunan Province, China between May 2019 and July 2019. A total of 2,030 healthcare workers were enrolled randomly in this study based on a three-stage stratified sampling method. The Neck Disability Index (NDI) was used to measure disability in subjects with neck pain. A self-administered questionnaire was used to assess the characteristics of individual and work organizational variables. The Mann–Whitney *U* test and the Kruskal–Wallis H test were applied to analyze the prevalence of pain intensity among groups. Multivariate linear regression analysis was performed to explore the potentional relationship between NSP and individual and work organizational variables using the NDI score as the dependent variable.

**Results:**

2,008 of 2,030 healthcare workers filled out the survey questionnaires online. In the past 12 months, 1,489 participants (74.2%) complained of pain in the cervical–shoulder region. NDI score increased by 0.10 for each year of age, with healthcare workers working in Obstetrics and Gynecology, and the Operating Room less likely to have NSP than those working in other departments. In contrast, female healthcare workers with a history of pregnancy were more likely to have NSP. In terms of organizational factors, workers who received concern from their supervisor about their health, who had the choice to change their shift status to off duty when they were not feeling well, or who were informed about WMSDs were less likely to have NSP.

**Conclusion:**

The prevalence of NSP within the previous year was high in this population. Individual factors including history of neck–shoulder diseases, age, and history of pregnancy and organizational factors including being adequately informed regarding WMSDs, concern from supervisors about workers' health, and the ability of workers to change their shift status to off duty when they were not feeling well were shown to induce significant effects to NSP.

## Introduction

Neck–shoulder pain (NSP) is defined as an subjective feeling of unpleasant from shoulder and neck region. Patients with NSP often complain about regional pain, numbness and other discomfort, with or without pain referred into head, torso, and upper limb regions (1). The growing prevalence of work-related musculoskeletal disorders (WMSDs) among healthcare workers has been called “the tip of an iceberg,” with a prevalence of 31.2 (2) to 88.0% (3), just secondary to lower back pain. NSP can be categorized by the degree of disability as mild, moderate, severe, very severe, and complete (4).

Negative impacts related to NSP have been grossly underappreciated. According to the Study of Global Burden of Disease, NSP is the fourth leading cause of disability, ranking behind back pain, depression (5), and arthralgias. Surgeons who experience neck pain have also been reported to be more likely to experience shoulder pain. Even in cases in which the pain is mild, repeated instances of pain can lead to repetitive strain injury, affecting the length of the surgeon's career (6).

Studies have shown that biological and physical factors such as age and lifestyle are associated with NSP (7), for example, it was reported that prevalence of neck pain reported at least once monthly in early adolescence was 38%, and genetic and environmental factors seem to play the most important roles in liability to neck pain (8). A survey conducted in Canada reported that more over than 80% of helicopter pilots and 85% of the crew-members had experienced neck pain caused by helicopter flights (9). But, to date, there has been limited research on the risk from organizational factors in healthcare settings. While education was not effective at preventing NSP (10), exercise was found to be effective (11). However, there was no evidence that it is beneficial to eliminate ergonomic or risk factors related to occupational neck–shoulder pain (ONSP). Over 60% of surgeon participating in one study experienced discomfort while performing vaginal procedures, with the most commonly affected body parts being the back, shoulders, and neck (12). High physical workload in surgeons was significantly associated with the risk of WMSDs in the trunk, longer duration procedures, and more severe fatigue (13). In a pre-experiment of the study on the relationship between working posture and WMSDs in registered nurses population, we found that the ratio of A3 and A4 were 15 and 21%, respectively in 45,825 valid images according to The Ovako Working posture Assessment System (OWAS)(14), which was similar to previous study (15). In the simulation of several routine task, the highest ratio of A3 and A4 (21 and 31%, respectively) appeared in the procedure of manual assisted to turn over patients' body and pat on the back. These findings indicate that persistent risk factors in the workplace may be associated with persistent pain and a poor prognosis for WMSDs.

Understanding the prevalence of and the relationship of individual and organizational factors with NSP would contribute to the development of intervention strategies that increase healthcare workers' understanding of how to prevent NSP and improve the workplace environment in medical institutions. Therefore, the aims of the current study were to investigate the characteristics of NSP and to identify the individual and organizational factors associated with it.

## Materials and Methods

### Ethical Approval and Consent to Participate

The Institutional Review Board of behavioral and nursing research at the School of Nursing at Central South University approved the study protocol (#2017033). Prior to collecting the data, written informed consent was obtained from each participant. The study was conducted in accordance with the Declaration of the World Medical Association and the Helsinki Declaration on the testing of human subjects.

### Trial Design and Tools

**Figure 1:**
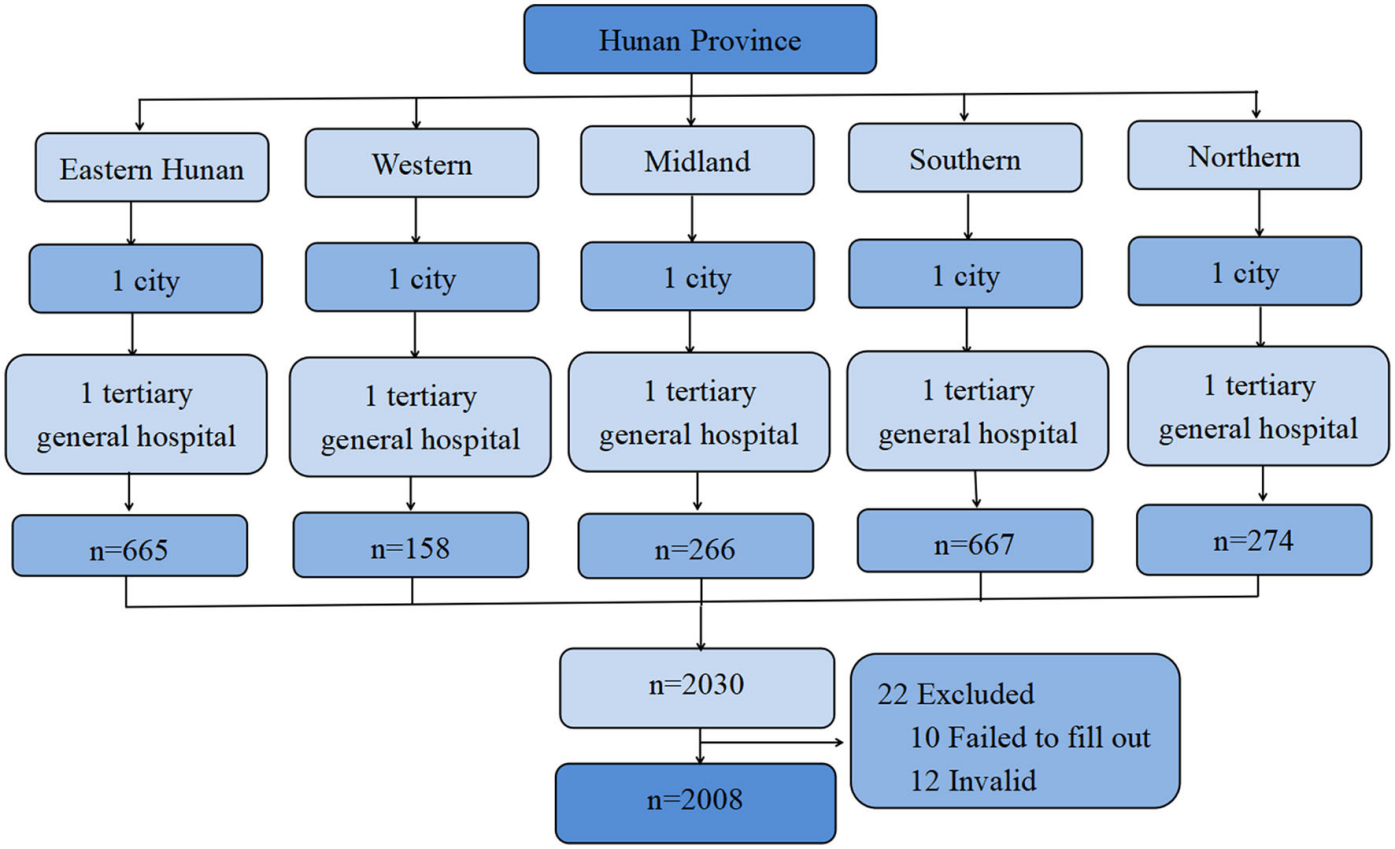
Flowchart of the multi-stage stratified sampling with random principle.

This cross-sectional study was performed in the tertiary general hospitals in Hunan Province, China between May 2019 and July 2019. Participants were recruited randomly based on a three-stage stratified sampling method: a flowchart of the sampling method is shown in Figure 1.

The Neck Disability Index (NDI), a self-administered questionnaire with 10 items, was used to measure disability in subjects with neck pain. The 10 items in this questionnaire measure pain intensity, personal care, lifting, reading, headache, concentration, work, driving, sleeping, and recreation. Each item is scored from 0 to 5 (for a maximum score of 50), and the higher the score, the greater the disability. The formula for the index of impaired neck function (INF) is: INF (%) = [total score from 10 items / (number of items completed × 5)] × 100. The criteria for the different INF ratings are 0–20%, 21–40%, 41–60%, 61–80%, and 81–100%, which, respectively represent mild, moderate, severe, very severe, and completely INF. Cronbach's α ranging from 0.74 to 0.93 have been reported for the NDI (16–18).

A further self-administered questionnaire was used to assess the characteristics of individual and work organizational variables. Based on human factors engineering theory, the survey questionnaire was compiled and then modified using two rounds of the Delphi method, in which 20 experts in the fields of occupational musculoskeletal injury, spinal rehabilitation, or human engineering evaluated its content and applicability. The authoritative coefficients of the two rounds of expert consultation were 0.92 and 0.93, respectively. The final version of the questionnaire had a Cronbach's α of 0.930, with each dimension having a Cronbach's α of between 0.680 and 0.924. The final questionnaire was composed of three part. Part one was basic information section including 12 items. Part two was the main body consisting of six dimensions: individual factors, facilities and equipment factors, workload factors, workspace and environment factors, and social psychological factors, which was comprised of 87 closed-ended questions. Part three was the morbidity of NSP including 10 items. In this study, we imposed the further requirement that the uncomfortable condition was recognized as NSP only when it continued for more than 1 week. We focused on the characteristics of NSP and the potentional relationship between the NSP and the individual and work organizational variables in this study.

### Participants

A total of 2,030 healthcare workers were enrolled in this study. The inclusion criteria were: (1) full-time registered physicians or registered nurses of 18–60 years of age; (2) employed as a clinician or nurse, with at least 1 year of clinical experience; (3) not received any medical therapy in the past 2 weeks; (4) taking part in this study voluntarily and cooperates well with investigators.

Subjects with any one of the following criteria were excluded: (1) diagnosed with cervical spondylotic myelopathy; (2) diagnosed with other severe diseases, such as diabetes mellitus, cardiovascular and cerebrovascular diseases, or tumors; (3) history of neck trauma, neck fracture, or neck surgery; (4) pregnant or breastfeeding women;(5)fibromyalgia syndromes.

### Data Collection

We distributed the questionnaire to administrative staff in the nursing department of selected hospitals via the “Questionnaire Star” online survey platform. The administrative staff then distributed the questionnaires to a special WeChat working group, which included staff from the following units: General Internal, General Surgical, Obstetrics and Gynecology, Pediatrics, Operating Room, and Intensive Care. Nurses completed the questionnaire by clicking on the survey link or the quick response (QR) code of the online questionnaires, which were forwarded by the administrative staff of their nursing department. Every participant was asked to read and consent to the agreement of informed consent before replying to the survey. The collection of questionnaire data was completed anonymously, without acquiring any personal details from the participants and on a voluntary basis. To increase the response rate, an honorarium of nearly $2 USD was paid to each respondent who completed the questionnaire.

### Statistical Analysis

SPSS Statistics version 23 (IBM Corp.) was used to perform all statistical analyses. Data were summarized as frequencies and percentages, or as means and standard deviations, as applicable. The Mann–Whitney *U* test and the Kruskal–Wallis H test were applied to analyze the prevalence of pain intensity among the groups. We performed multivariate linear regression analysis to explore risk factors, with the NDI score as the dependent variable, and the potential individual and organizational factors related to NSP as the independent variables. The odds ratio (OR) and 95% confidence interval (CI) were estimated from the multivariate regression analysis. To reduce confounding bias, we performed a backward linear regression analysis (α entry = 0.10, α removal = 0.15). A two-tailed *P* < 0.05 was considered statistically significant for all tests.

## Results

### Population Characteristics

In total, 2,008 of 2,030 healthcare workers filled out the survey questionnaires online with no missing data, giving an overall response rate of 99.41%. An overview of the participants is provided in Table 1. In this data set, 537 participants (26.74%) were clinicians, and the rest (73.26%) were clinical nurses. The number of participants from each group varied by region, with this variability being due to the difference in sizes of the selected hospitals. In terms of the prevalence of pain intensity, there was no significant regional difference among the five groups (Eastern, Southern, Northern, Central, and Western) (*P* for trend = 0.075).

**Table 1:** Demographic characteristics and prevalence of neck-shoulder pain in 2,008 participants.

**Items**			**INF rating**			***P*** **-value**
	**Mild**	**Moderate**	**Severe**	**Very severe**	**Total (%)**	
	**Participants (%)**	**Participants (%)**	**Participants (%)**	**Participants (%)**	**Participants (%)**	
**City**						
Eastern	433 (65.11)	203 (30.53)	26 (3.91)	3 (0.45)	665 (100)	0.08
Southern	101 (69.18)	37 (25.34)	8 (5.48)	0 (0.00)	146 (100)	
Northern	158 (60.54)	93 (35.63)	10 (3.83)	0 (0.00)	261 (100)	
Central	402 (60.27)	228 (34.18)	35 (5.25)	2 (0.30)	667 (100)	
Western	185 (68.77)	67 (24.91)	15 (5.58)	2 (0.74)	269 (100)	
**Clinical department**						
General internal unit	443 (65.44)	199 (29.39)	34 (5.02)	1 (0.15)	677 (100)	0.05
General surgical unit	325 (61.90)	163 (31.05)	32 (6.10)	5 (0.95)	525 (100)	
Obstetrics and gynecology	113 (70.63)	44 (27.50)	3 (1.88)	0 (0.00)	160 (100)	
Pediatric unit	126 (63.64)	66 (33.33)	5 (2.53)	1 (0.51)	198 (100)	
Operating room	139 (64.95)	67 (31.31)	8 (3.74)	0 (0.00)	214 (100)	
Intensive care unit	133 (56.84)	89 (38.03)	12 (5.13)	0 (0.00)	234 (100)	
**Occupation**						
Clinician	329 (61.27)	177 (32.96)	29 (5.40)	2 (0.37)	537 (100)	0.15
Clinical nurse	950 (64.58)	451 (30.66)	65 (4.42)	5 (0.34)	1,471 (100)	
**Health technique title**						
Nurse	294 (79.25)	69 (18.60)	8 (2.16)	0 (0.00)	371 (100)	0.00
Nurse Practitioner	422 (63.65)	210 (31.67)	28 (4.22)	3 (0.45)	663 (100)	
Nurse-in-charge	193 (52.59)	145 (39.51)	27 (7.36)	2 (0.54)	367 (100)	
Associate Professor of Nursing	38 (59.38)	24 (37.50)	2 (3.13)	0 (0.00)	64 (100)	
Professor of Nursing	3 (50.00)	3 (50.00)	0 (0.00)	0 (0.00)	6 (100)	
Doctor	150 (70.75)	48 (22.64)	13 (6.13)	1 (0.47)	212 (100)	
Doctor-in-charge	118 (51.98)	97 (42.73)	11 (4.85)	1 (0.44)	227 (100)	
Associate Professor of Medicine	52 (58.43)	32 (35.96)	5 (5.62)	0 (0.00)	89 (100)	
Professor of Medicine	9 (100.00)	0 (0.00)	0 (0.00)	0 (0.00)	9 (100)	
**Marital status**						
Unmarried	403 (73.94)	128 (23.49)	14 (2.57)	0 (0.00)	545 (100)	0.00
Married	855 (59.79)	493 (34.48)	75 (5.24)	7 (0.49)	1,430 (100)	
Live apart	3 (60.00)	0 (0.00)	2 (40.00)	0 (0.00)	5 (100)	
Divorced	18 (64.29)	7 (25.00)	3 (10.71)	0 (0.00)	28 (100)	
**History of pregnancy**						
No	511 (73.21)	169 (24.21)	17 (2.44)	1 (0.14)	698 (100)	0.00
Yes	768 (58.63)	459 (35.04)	77 (5.88)	6 (0.46)	1,310 (100)	
**Education**						
Junior college degree	292 (66.67)	129 (29.45)	17 (3.88)	0 (0.00)	438 (100)	0.19
Undergraduate degree	898 (62.40)	468 (32.52)	67 (4.66)	6 (0.42)	1,439 (100)	
Graduate degree	89 (67.94)	31 (23.66)	10 (7.63)	1 (0.76)	131 (100)	
**Income (**¥**/year)**						
<3,000	120 (76.92)	25 (16.03)	11 (7.05)	0 (0.00)	156 (100)	0.01
3,000–5,000	497 (62.99)	253 (32.07)	36 (4.56)	3 (0.38)	789 (100)	
5,000–8,000	484 (60.80)	270 (33.92)	38 (4.77)	4 (0.50)	796 (100)	
8,000–10,000	139 (67.48)	59 (28.64)	8 (3.88)	0 (0.00)	206 (100)	
>10,000	39 (63.93)	21 (34.43)	1 (1.64)	0 (0.00)	61 (100)	
**Gender**						
Male	157 (58.00)	93 (34.83)	16 (5.99)	1 (0.37)	267 (100)	0.06
Female	1,122 (64.45)	535 (30.73)	78 (4.48)	6 (0.34)	1,741 (100)	
**Age (years)**						
<25	243 (82.37)	46 (15.59)	6 (2.03)	0 (0.00)	295 (100)	0.00
25–35	724 (63.34)	355 (31.06)	60 (5.25)	4 (0.35)	1,143 (100)	
35–45	236 (52.68)	187 (41.74)	23 (5.13)	2 (0.45)	448 (100)	
45–55	70 (64.22)	33 (30.28)	5 (4.59)	1 (0.92)	109 (100)	
>55	6 (46.15)	7 (53.85)	0 (0.00)	0 (0.00)	13 (100)	
**Height (cm)**						
<155	68 (64.15)	35 (33.02)	3 (2.83)	0 (0.00)	106 (100)	0.01
155–160	419 (65.88)	185 (29.09)	30 (4.72)	2 (0.31)	636 (100)	
160–165	458 (61.98)	239 (32.34)	40 (5.41)	2 (0.27)	739 (100)	
165–170	214 (69.71)	83 (27.04)	8 (2.61)	2 (0.65)	307 (100)	
170–175	74 (55.64)	56 (42.11)	3 (2.26)	0 (0.00)	133 (100)	
>175	46 (52.87)	30 (34.48)	10 (11.49)	1 (1.15)	87 (100)	
**Weight (kg)**						
<45	64 (72.73)	21 (23.86)	3 (3.41)	0	88 (100)	0.03
45–55	517 (65.11)	243 (30.60)	32 (4.03)	2 (0.25)	794 (100)	
55–65	477 (63.77)	222 (29.68)	45 (6.02)	4 (0.53)	748 (100)	
65–75	183 (60.20)	114 (37.50)	6 (1.97)	1 (0.33)	304 (100)	
>75	38 (51.35)	28 (37.84)	8 (10.81)	0 (0.00)	74 (100)	
**Body mass index**						
18.5– <25	1,128 (64.53)	534 (30.55)	79 (4.52)	7 (0.40)	1,748 (100)	0.14
25– <30	145 (58.23)	89 (35.74)	15 (6.02)	0 (0.00)	249 (100)	
30– <40	6 (54.55)	5 (45.45)	0 (0.00)	0 (0.00)	11 (100)	

*INF, impaired neck function*.

### Prevalence of NSP in Healthcare Workers

**Figure 2:**
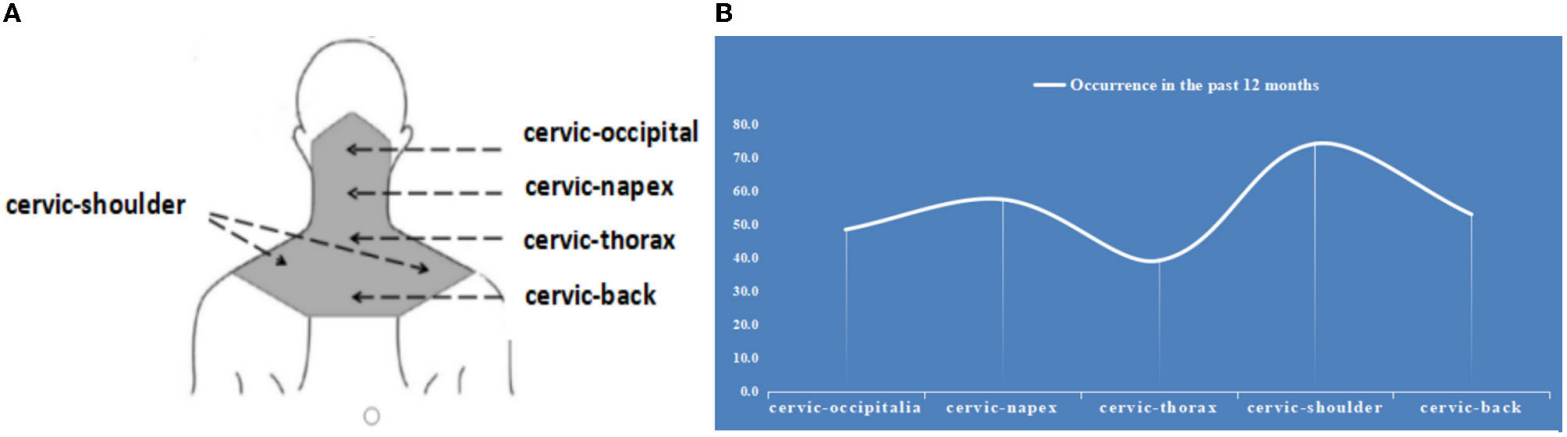
Prevalence of chronic neck shoulder pain in 2,008 participants in the past 12 months. **(A)** Diagram of neck region. **(B)** Prevalence of shoulder and neck pain in 2008 participants in the past 12 months.

The distribution of the anatomical sites of NSP in this study are shown in Figure 2A. In the 12 months prior to the questionnaire, only 6.5% participants reported no NSP, while 1,489 participants (74.2%) complained of pain in the cervical–shoulder region. This percentage was far higher than those in the cervical–occipital, cervical–napex, cervical–thoracic, and cervical–back regions (Figure 2B). In this data set, only 291 participants (14.49%) reported their NSP to their hospital. There were 143 cases (7.12%) in which participants were forced to change their shift due to the NSP.

**Figure 3:**
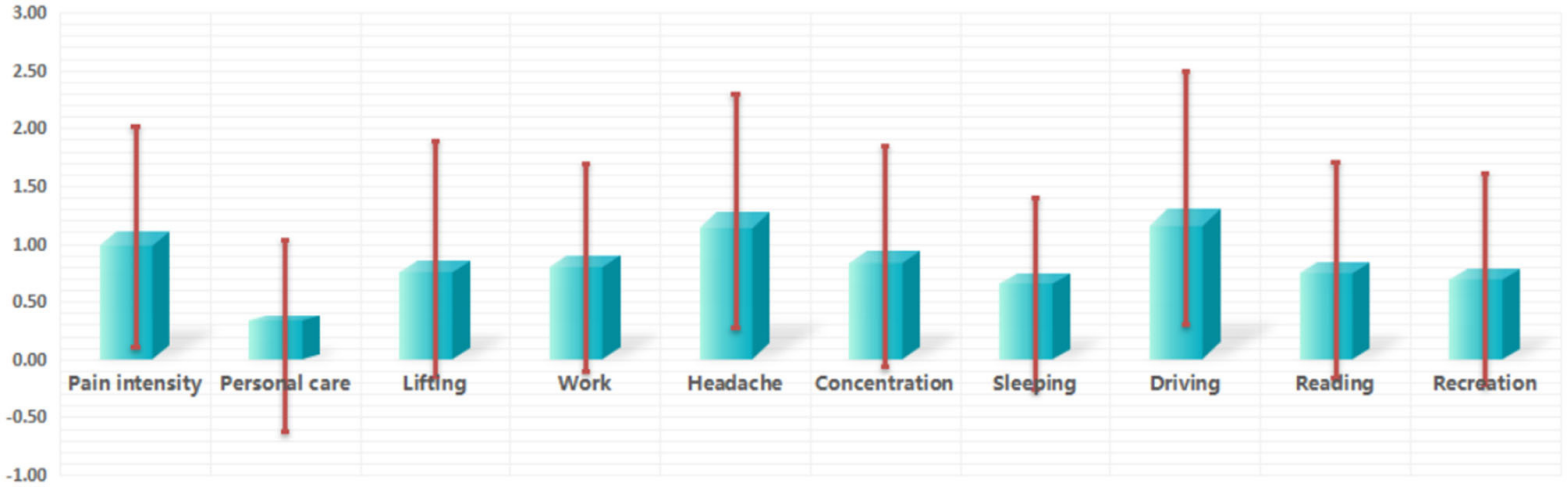
Item-total score of the NDI.

With respect to the prevalence of pain intensity in this study, no participants were categorized as having complete INF, while the numbers of participants with mild, moderate, severe, and very severe INF, respectively were 1,279 (63.70%), 628 (31.27%), 94 (4.68%), and 7 (0.35%). Of the 10 items on the NDI, the “driving” item scored the highest (1.30 ± 1.20), with “headache” (1.28 ± 1.02) coming second (Figure 3).

### Individual Factors Affecting the Severity of NSP in Healthcare Workers

A non-parametric test revealed a significant difference in the prevalence of pain intensity among the different age groups (*P* for trend = 0.00), while linear regression analysis further revealed that the NDI score increased by 0.10 with each additional year of age (OR = 0.10; 95% CI: 0.03 to 0.16; *P* = 0.00). Furthermore, healthcare workers from the Obstetrics and Gynecology (OR = −1.64; 95% CI: −2.76 −0.52; *P* = 0.01) or Operating Room (OR = −1.51; 95% CI: −2.61 – −0.40; *P* = 0.01) units were less likely to experience NSP than those from other departments. In terms of their history of pregnancy, 354 of 1,122 women (31.55%) had not experienced pregnancy, but those who had were more likely to experience NSP (OR = 1.03; 95% CI: −0.20 – 2.27; *P* = 0.00). Participants with a history of neck–shoulder diseases were more likely to have NSP than those without this history (OR = 1.31; 95% CI: 0.22–2.38; *P* = 0.02). In terms of personal daily living habits, habitually awkward postures (OR = 0.92; 95% CI: 0.34–1.49; *P* = 0.00) and sleeping with a relatively high pillow (OR = 0.89; 95% CI: 0.08–1.71; *P* = 0.03) were risk factors for a higher NDI score.

Interestingly, reading or watching the TV or computer in a reclining posture were protective factors resulting in a lower prevalence of NSP in healthcare workers (OR = −0.84; 95% CI: −1.36– −0.32; *P* = 0.00). Additionally, participants who felt a greater sense of tiredness after housework were more likely to experience NSP (OR = 1.43; 95% CI: 0.99–1.86; *P* = 0.00) (Table 2).

**Table 2:** Factors related to neck-shoulder pain in healthcare workers: linear regression analysis.

**Variables**	**Unstandardized coefficient**		**Standarized coefficient**	***t***	***P***	**95% CI**
	**β**	**Std. error**	**Beta**			
Age (years)	0.10	0.03	0.11	3.00	0.00	0.03–0.16
Body height (cm)	0.06	0.04	0.06	1.79	0.07	−0.01–0.13
History of pregnancy	1.03	0.63	0.06	1.64	0.00	−0.20–2.27
Obstetrics and gynecology	−1.64	0.57	−0.10	−2.87	0.01	−2.76– −0.52
Pediatric unit	−0.96	0.57	−0.06	−1.70	0.09	−2.08–0.15
Operating room	−1.51	0.56	−0.10	−2.68	0.01	−2.61– −0.40
Occupation	0.72	0.49	0.06	1.46	0.14	-0.24–1.68
History of neck–shoulder diseases	1.31	0.55	0.08	2.37	0.02	0.22–2.38
Habitually awkward postures during daily life	0.92	0.29	0.12	3.11	0.00	0.34–1.49
Habit of using a high pillow during sleep	0.89	0.42	0.07	2.14	0.03	0.08–1.71
Degree of tiredness felt after housework	1.43	0.22	0.22	6.44	0.00	0.99–1.86
Acquired training regarding WMSDs	−0.70	0.21	−0.12	−3.29	0.00	−1.11– −0.28
Received concern from supervisor about health	−0.51	0.22	−0.09	−2.33	0.02	−0.94– −0.08
Ability to change shift status to off duty when they are not feeling well	−0.89	0.22	−0.15	−4.03	0.00	−1.32– −0.45

*R-square = 0.182; F = 10.501, P = 0.00*.

### Organizational Factors Affecting the Severity of NSP in Healthcare Workers

Ten items were included in the questionnaire assessing organizational factors, four of which entered the final regression model. Healthcare workers had a higher risk of NSP if their professional department did not deal with their WMSD concerns well (OR = 0.55; 95% CI: 0.89–1.86; *P* = 0.00). Additionally, the ability to change their shift status to off duty when they were not feeling well (OR = −0.89; 95% CI: −1.32– −0.45; *P* = 0.00), concern from their supervisor about their health (OR = −0.51; 95% CI: −0.94– −0.08; *P* = 0.02), and receiving training regarding WMSDs (OR = −0.70; 95% CI: −1.11– −0.28; *P* = 0.00) were also protective factors (Table 2).

## Discussion

This cross-sectional study of NSP in healthcare workers revealed the following: (1) the prevalence of NSP within the previous year was high in this population, with pain in the cervical–shoulder region being the most common; (2) the individual factors associated with NSP were a history of neck–shoulder diseases, age, and a history of pregnancy; (3) the organizational factors linked with NSP were that the healthcare worker had acquired adequate training regarding WMSDs, had received concern from their supervisor about their health, had the ability to change their shift status to off duty when they were not feeling well, and could deal with WMSD complaints via their professional department and *via* experts; (4) the extent to which participants felt tired after housework and a habit of sleeping with a high pillow were predictors of NSP, while reading or watching the TV or computer in a reclining posture reduced the likelihood of NSP; (5) participants working in the ICU were more likely to experience NSP compared with those from other units, including the Obstetrics and Gynecology or Operating Room units.

We demonstrated that the prevalence of NSP within the previous year was high in healthcare workers in China and that pain in the cervical–shoulder region was more common than that in the cervical–occipital, cervical–napex, cervical–thoracic, and cervical–back regions. Previous studies have reported 1-year prevalence estimates of neck pain in office workers ranging from 42–69% (19–21). However, we observed a prevalence of up to 90% in our study, which was far higher than that in office worker population samples and the general population with an annual prevalence rate exceeding 30% (22). It is also worth noting that approximately three-quarters of participants experienced pain in the cervical-shoulder region, followed by pain in the cervical-napex and cervical-back regions. Healthcare workers usually spend less time sitting compared with workers in other occupations, but perform a wide range of healthcare activities and procedures, involving repetitive movements, forceful procedures, highly demanding work, work in a static posture, and exposure to vibration. Some ergonomists have described the working conditions of healthcare workers as being equal to those of certain industrial workers (23). Therefore, a deeper exploration of the risk factors linked to NSP in healthcare workers is warranted, suggesting that randomized controlled study should be perfomed and data on some characteristics of the work performed could be collected in the future.

In terms of the individual factors associated with NSP, we confirmed that age has a significant impact on NSP, in line with previous publications (24). Functional and structural changes in the musculature surrounding the spine and the intervertebral disc with increasing age are theorized to lead to a significant impact on passive spine stiffness and discomfort responses (24). With respect to the association between a history of neck–shoulder diseases and NSP, the local muscles of individuals with such diseases may fatigue more easily during work than those of others, which was similar to previous studies. Of note, we found that women with a history of pregnancy were more likely to experience NSP than those with no such history. The age of participants in the current study was concentrated in the range between 25 and 45 years, with approximately one-third of women having never been pregnant. The prevalence of neck pain has been reported to be associated with gender, with females having a higher prevalence than males (25). Pregnancy-induced biomechanical, hormonal, and vascular changes are likely to give rise to a wide variety of musculoskeletal problems (26–28). Spinal pain has been reported as the most frequent disorder during pregnancy. Positive associations have been found between low back pain and pelvic pain and pregnancy due to altered pelvic joint mechanics and/or altered muscular function (29, 30). However, whether hormonal and vascular changes induced by pregnancy can lead to long-term musculoskeletal problems in individuals is unknown. Therefore, future research should be directed to identifying pathways for pregnancy influence on NSP and in exploring the mechanism whether neuropathic or mechanical.

Interestingly, in terms of the organizational factors linked to NSP, we found that participants working in the ICU were more likely to experience NSP than those working in other units, including the Obstetrics and Gynecology or Operating Room units. It might because of what healthcare workers in the ICU have to provide more bedside nursing procedures involving a static awkward posture, such as oral care, sputum suction and the procedure of manual assisted to turn over patients' body and pat on the back. This hypothesis was confirmed in our subsequent simulation experiment. Most of the subjects in this experiment complained of extreme shoulder and neck discomfort, with RPE scores up to 9. Hence, we recommend that nursing managers review our nursing procedures and optimize them to minimize the frequency and duration of awkward postures. In this study, participants responded that they would have less risk of NSP if they received more concern from their supervisor, had the ability to change their shift status to off duty when they were not feeling well, and could undertake more training regarding WMSDs. This means it's needed for healthcare seetings to develop a series of action programme to help their employees and make ensure their health. Therefore, our results indicate that the importance of organizational factors in the development of NSP might have been underestimated.

In addition, our results revealed that feeling more tired after housework and the habit of having a high pillow during sleep were potential predictors of NSP in healthcare workers. In contrast, participants who read or watched the TV or computer in a reclining posture were less likely to experience NSP, suggesting that this posture offers the opportunity to release the muscles in their shoulders and neck.

### Limitations

There are several clinical and research implications that follow from our study. Factors including a history of neck–shoulder diseases, age, acquiring training about WMSDs, and receiving concern from supervisors were significantly associated with NSP. For clinicians and policy makers, our study draws attention to healthcare workers with NSP, as we revealed several common factors and potential predictors of such pain. However, to our knowledge, no prior studies have reported a link between a history of pregnancy and NSP, and additional prospective studies will be necessary to further clarify this matter. Of course, our study also had several limitations. We included 2,008 participants from one province in China, which may have led to a sampling bias due to the regional nature of the study. Second, we collected data from an online questionnaire using self-reported measures, not objective parameters for the assessment of disability and pain, and this might lead to the result with more subjective. Third, due to the cross-sectional nature of the study, the main limitation was the absence of a control group, which may lead to the exact causal relationship cannot be established. Finally, observational method was not used in this study, this lead to the lack of data on some characteristics of the work performed.

## Data Availability Statement

The original contributions presented in the study are included in the article/supplementary material, further inquiries can be directed to the corresponding author.

## Ethics Statement

The studies involving human participants were reviewed and approved by The Institutional Review Board of behavioral and nursing research at the School of Nursing at Central South University approved the study protocol (#2017033). The patients/participants provided their written informed consent to participate in this study.

## Author Contributions

SY and HW contributed in conception, study design, coordination of data collection, and acquisition in data. SY, YL, and LZ were responsible for interpretation of data, drafting, and writing and finishing the manuscript. All authors read and approved the final manuscript.

## Funding

This study was supported by the Foundation of Innovation Project of Science and Technology of Hunan Province (Grant No. 2017SK50107).

## Conflict of Interest

The authors declare that the research was conducted in the absence of any commercial or financial relationships that could be construed as a potential conflict of interest.

## Publisher's Note

All claims expressed in this article are solely those of the authors and do not necessarily represent those of their affiliated organizations, or those of the publisher, the editors and the reviewers. Any product that may be evaluated in this article, or claim that may be made by its manufacturer, is not guaranteed or endorsed by the publisher.
